# Ultra-deep mutant spectrum profiling: improving sequencing accuracy using overlapping read pairs

**DOI:** 10.1186/1471-2164-14-96

**Published:** 2013-02-12

**Authors:** Haiyin Chen-Harris, Monica K Borucki, Clinton Torres, Tom R Slezak, Jonathan E Allen

**Affiliations:** 1 Lawrence Livermore National Laboratory, 7000 East Avenue, Livermore, CA, USA

**Keywords:** Quasispecies, Viral evolution, DNA mutational analysis, High-throughput sequencing, Diagnostics, Biomarker, Rare mutations, Sequencing error correction, Overlapping read pairs

## Abstract

**Backgound:**

High throughput sequencing is beginning to make a transformative impact in the area of viral evolution. Deep sequencing has the potential to reveal the mutant spectrum within a viral sample at high resolution, thus enabling the close examination of viral mutational dynamics both within- and between-hosts. The challenge however, is to accurately model the errors in the sequencing data and differentiate real viral mutations, particularly those that exist at low frequencies, from sequencing errors.

**Results:**

We demonstrate that overlapping read pairs (ORP) -- generated by combining short fragment sequencing libraries and longer sequencing reads -- significantly reduce sequencing error rates and improve rare variant detection accuracy. Using this sequencing protocol and an error model optimized for variant detection, we are able to capture a large number of genetic mutations present within a viral population at ultra-low frequency levels (<0.05%).

**Conclusions:**

Our rare variant detection strategies have important implications beyond viral evolution and can be applied to any basic and clinical research area that requires the identification of rare mutations.

## Background

Viruses with RNA genomes replicate with extremely high mutation rates because their RNA polymerases lack the proofreading ability of DNA polymerases. With a mutation rate of ~1 error per 10,000 nucleotides copied, a point mutation is introduced nearly every time a single RNA virus replicates [1]. Any given viral sample extracted from a host contains a spectrum of related genotypes, referred to as a quasispecies, whose ability to rapidly evolve underlies viral virulence, vaccine resistance and host-jumping [2]. Understanding the mutational dynamics of RNA viruses is key to our understanding of viral disease progression, transmission and the development of antiviral therapeutics.

Considerable progress has been made recently using deep sequencing to characterize the mutant spectra in several human RNA viral pathogens: human immunodeficiency virus (HIV) [3-6], hepatitis C virus (HCV) [7-9] and influenza [10,11]. In particular, deep sequencing has been used to identify medically relevant drug-resistant rare variants that impact anti-retroviral drug treatment outcomes [11-15]. Many of the earliest efforts have targeted specific hyper variable genomic regions using 454 sequencing; more recently published studies have begun to target more of the genome with high depths using the greater sequencing output of Illumina technologies [16,17]. Recently, the software tool for managing deep sequencing data, Segminator II, was introduced and used to compare performance of Illumina and 454 deep sequencing of influenza [18]. However, less emphasis was given to evaluating a general subconsensus base calling procedure and the impact of PCR amplification was not considered. Two other available packages that address PCR errors and known sequencing error modes, AmpliconNoise [19] and RC454 [20], are designed specifically for 454 pyrosequencing data. In a recent Illumina deep sequencing study of foot-and-mouth disease virus samples, Wright et al. [21] counted evidence for between 1,434 and 2,622 rare variants present in their samples. Their approach relied on an estimated error rate from the sequencer without the use of sequencing controls and included sequencing each sample twice to correct for sequencing error, which would present practical problems for sequencing larger numbers of samples collected from an outbreak. Moreover, recent work has indicated the presence of non-uniform error rates in Illumina sequence data in particular, and highlights the ongoing challenge of correctly separating the true mutant spectra from sequencing related errors [15,22-24].

To date, few investigations have applied high throughput sequencing on viruses that naturally circulate in animal hosts. Given the potential for RNA viruses that circulate in a non-human host reservoir to infect new host types, including humans [25], it is important to study viral evolution at the highest resolution for measuring genetic change. Characterizing these viruses pose a particular challenge since the starting material can be too small (or rare) to directly sequence without PCR amplification or growth in cell culture, which can introduce new errors to the measurement process [26]. As a pilot study for a series of viral evolution studies, three viral samples collected from naturally infected hosts--two fox rabies brain tissue samples and one bovine coronavirus (BCV) nasal sample–were sequenced using Illumina paired-end read technology at ultra-deep coverage (> 300,000x raw reads). Two plasmid clones containing 1 kb region of the rabies and BCV genomes served as error controls in the study. Error rates seen in plasmids provided a best estimate of the combined PCR and sequencing error rate for the natural samples [27].

Overlapping paired-end reads, generated using short sequencing fragment libraries combined with long read lengths, have been recently used to improve Illumina paired-end read assemblies in software packages such as FLASH [28] and PANDAseq [29], in which overlapping regions of the read pairs serve to extend read lengths and reduce sequencing errors. Overlapping read pairs have not been previously applied to mutant spectra profiling to improve the sensitivity of variant detection. We demonstrate the novel use of mismatch rates in the overlapping read pairs (ORP) to provide an unbiased assessment of the sequencer-derived quality scores when selecting a read filtering threshold, as well as to estimate position-dependent sequencing errors without relying on a clonal control. Our methods identified a high variation in error rates between sequencers and indicated the difficulty of relying on traditional sequencing approaches for rare variant characterization. Moreover, we demonstrate that PCR amplification can become the dominant source of error over the sequencer’s error even when using a high fidelity polymerase. Among the three natural viral samples we sequenced, we identified up to 2,133 rare variants within a single sample with an in-host population frequency as rare as 0.0096%. Given the majority of the variants we discovered occurred at the ultra-rare level (< 0.1%), we show that careful error control and estimation using ORP can reveal a deep and rich mutant spectrum. Our results demonstrate a practical sequencing and computational analytic approach to studying viral evolution with an unprecedented level of genetic resolution.

## Results and discussion

### ORPs reduce error rates and provide benchmarks for quality scores

**Figure 1:**
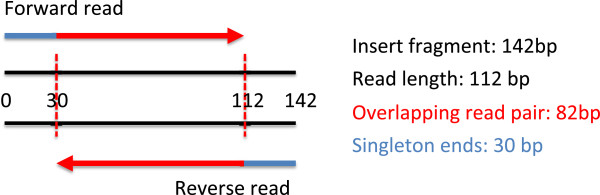
**Example of an 82-base overlapping read pair (in red).** Read length is 112 and the insert fragment length is 142. Black lines denote the double stranded DNA insert fragment, numbers between the black lines denote positions in the sequence (from 1 to 142).

For all five samples, 3 natural viral and 2 plasmid controls, Illumina paired-end sequencing was carried out using relatively short sequencing fragment libraries combined with relatively long reads (112 bp) to generate overlapping read pairs (ORP). The target DNA fragment in the library preparation was 142 bp. The resulting paired-end reads had an average overlap of 88 bp. Figure 1 illustrates the typical overlap of two paired-end reads. Table 1 and Additional file 1: Table S1 summarize the sequencing output and coverage levels for each read type – raw, ORP and singleton ends -- for each sample.

**Table 1:** Sequence coverage for the three natural RNA viral samples and the two plasmid control samples

	**Rabies Fox1**	**Rabies Fox2**	**BCV**	**BCV-Control**	**Rabies-Control**
No. bases sequenced	10,905 bp	11,183 bp	13,434 bp	1,012 bp	1,128 bp
Raw/single read coverage	518,930	522,347	356,027	732,294	4,306,793
ORP (Q≥30) coverage	80,546	96,198	79,037	128,943	773,021
Singleton ends coverage	139,599	114,217	69,584	176,087	1,098,167

Mean coverage per base is shown for raw reads (single, unpaired reads), overlapped regions of read pairs and singleton ends portions of the read pairs. Coverage for overlapping read pairs (ORP Q**≥30)** are reported in the unit of pairs in this table, to reflect that the two strands in the pair report redundant information on one insert fragment.

The long overlapping regions in the read pairs offered several important practical benefits to improving the quality of the reads. First, they served as a mechanism of error checking, as each read pair came from the same template and should therefore be perfectly complementary. Base calls that do not match in the forward and reverse strands are automatically identified as sequencing errors. Second, although the mismatched bases were excluded from data analysis, they provided an empirical estimate for single-read sequencing error rates. Third, it identified “problematic loci” on the genome where large fractions of the ORPs are mismatched. A high fraction of ORP mismatches at a particular locus would indicate that the locus was a site with high probability of erroneous nucleotide incorporation and hence suggest that a more stringent criterion should be considered when making variant calls at the locus.

**Figure 2:**
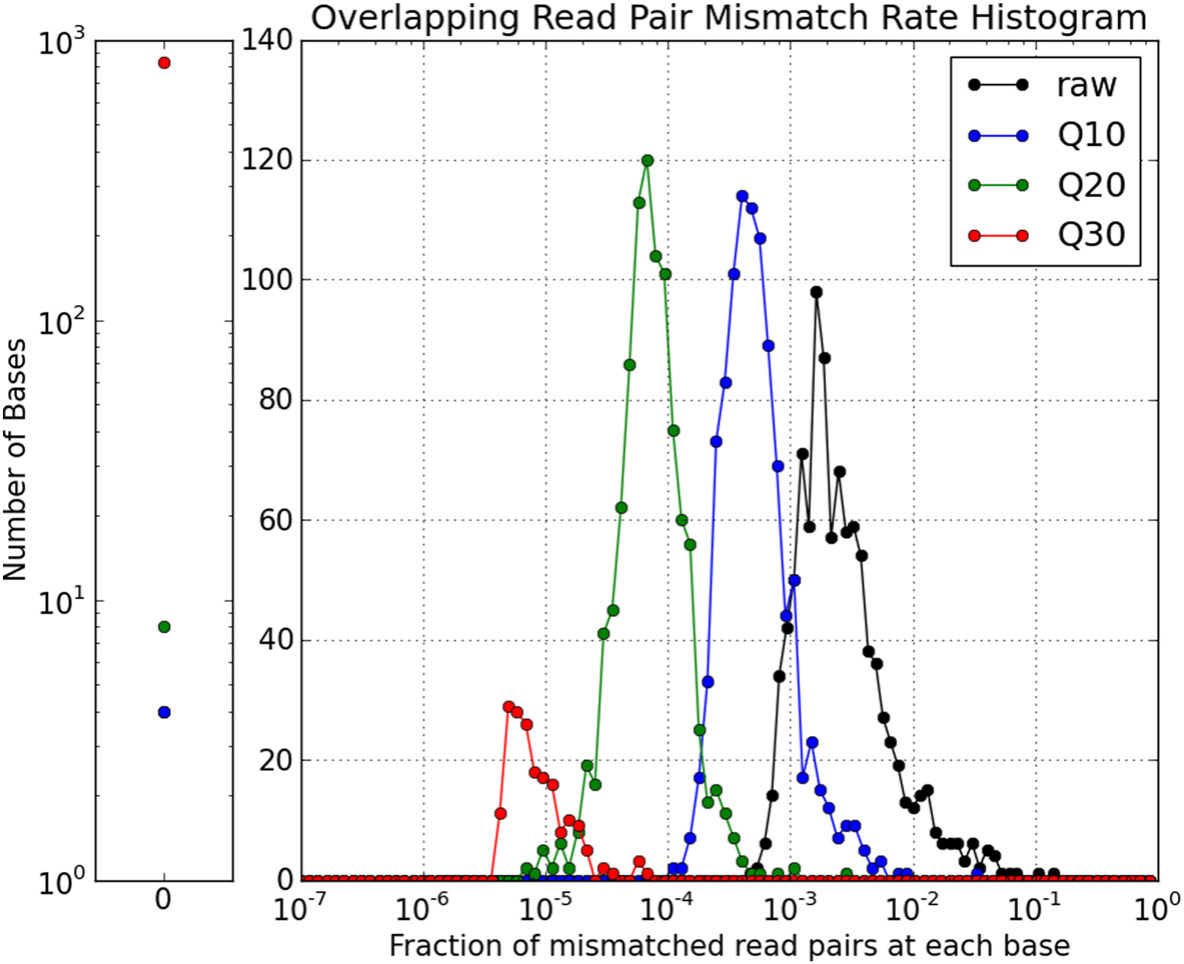
**Histogram of ORP mismatch rate (per base) in the BCV control sample.** Histograms are compiled for four different read populations: raw, unfiltered ORP reads (black), ORP reads with Q**≥**10, Q**≥**20 and Q**≥**30 shown in blue, green and red, respectively. Left plot shows the number of bases where mismatch frequency is zero for the four read populations. Both the raw and Q10 read pools had 4 bases where the mismatch frequency is zero (black and blue dots on the left panel overlap). Both left and right plots have number of bases as the ordinate.

The relationship between quality score – Phred like scores assigned by Illumina sequencers -- and the ORP mismatch frequency is illustrated in Figure 2 for the BCV control data. The fraction of mismatched read pairs at each base dramatically decreased with increasing Q-score. The number of positions in the 1 kb genome where no mismatches occurred jumped from 4 at Q10 to 828 at Q30 (Figure 2 left panel). The probability of a mismatched read pair occurring anywhere during the Illumina paired-end sequencing process was estimated from the ratio of number of mismatched ORPs over the total number of ORPs. These average ORP mismatch rates are shown in Table 2. While mismatches occurred at the approximate rate of ~7 per 1000 unfiltered base pairs; with Q30 filtering, the mismatch rate dropped three orders of magnitude to ~4 per 1 million base pairs (averaged over 5 samples). Hence the probability of two sequencing errors occurring at the same read pair is extremely small.

**Table 2:** Overall mismatch rates in overlapping paired-end reads for raw read pairs, read pairs that have quality scores Q10, Q≥20, Q≥30 and Q≥35

**Sample**	**Raw**	**Q10 (1**×**10**^**-1**^**)**	**Q20 (1**×**10**^**-2**^**)**	**Q30 (1**×**10**^**-3**^**)**	**Q35 (3**×**10**^**-4**^**)**
BCV control	5.55×10^-3^	7.82×10^-4^	9.56×10^-5^	1.94×10^-6^	4.26×10^-8^
Rabies control	7.33×10^-3^	9.64×10^-4^	1.12×10^-4^	2.59×10^-6^	1.31×10^-7^
BCV	4.91×10^-3^	7.87×10^-4^	1.06×10^-4^	2.55×10^-6^	1.51×10^-7^
Fox1	8.92×10^-3^	1.01×10^-3^	1.73×10^-4^	8.82×10^-6^	6.92×10^-7^
Fox2	6.69×10^-3^	8.33×10^-4^	1.22×10^-4^	4.16×10^-6^	2.28×10^-7^
Average of 5 samples	6.68×10^-3^	8.75×10^-4^	1.22×10^-4^	4.01×10^-6^	2.49×10^-7^
Rabies control –repeat run	5.99×10^-3^	2.90×10^-3^	1.04×10^-3^	2.67×10^-4^	8.00×10^-5^

The rabies control plasmid was sequenced a second time using a separate protocol at a different service provider and listed here for comparison. Mismatch rates are ‘per base pair’ so that they can be compared directly across samples and platforms. Phred error rates for the Q scores are shown in the heading.

The Q-score associated with each base-call is derived based on an aggregate of sequencer metrics and sample characteristics measured during each sequencing run. Q-scores of 10, 20, 30 and 35 correspond to error rates of 0.1, 0.01, 0.001, 0.0003, respectively. The mean mismatch rates among the raw, Q10, Q20, Q30 and Q35 ORPs were 6.7×10^-3^, 8.8×10^-4^, 1.2×10^-4^, 4.0×10^-6^, 2.5×10^-7^, respectively (Table 2, average of 5 samples), far lower than what their Illumina Q-scores suggest. However, when the rabies control plasmid was sequenced a second time, the resulting ORP mismatch rate profile was quite different. Though the mismatch rates were comparable to the first run in the raw, unfiltered ORP reads, they were significantly higher in the second run when comparing the same Q thresholds (Table 2). At Q30, ORP mismatch rates in the first run were two orders of magnitude lower than the rabies control repeat run. Given the same model of Next Generation Sequencing instrument (Illumina GA IIx) was used in both sequencing runs, the most parsimoneous explanation for the difference in ORP mismatch rates is the difference in the Q-score calibration of the two instruments. This underscores the utility of using ORP to recover an empirical sequencing error rate and minimize technical artifacts introduced by the sequencer.

The trade-off for enhanced accuracy is the reduction in coverage. Since the two overlapping reads represent redundant information from the same amplicon, the true depth of coverage is the number of read pairs instead of number of single reads. For example, in the BCV control sample, the Q-score filtering process removes roughly 11%, 22% and 55% of the raw matching ORPs at each base at Q10, Q20 and Q30, respectively (see Additional file 1: Figure S1).

### ORPs help distinguish PCR error rates from sequencing error rates

**Figure 3:**
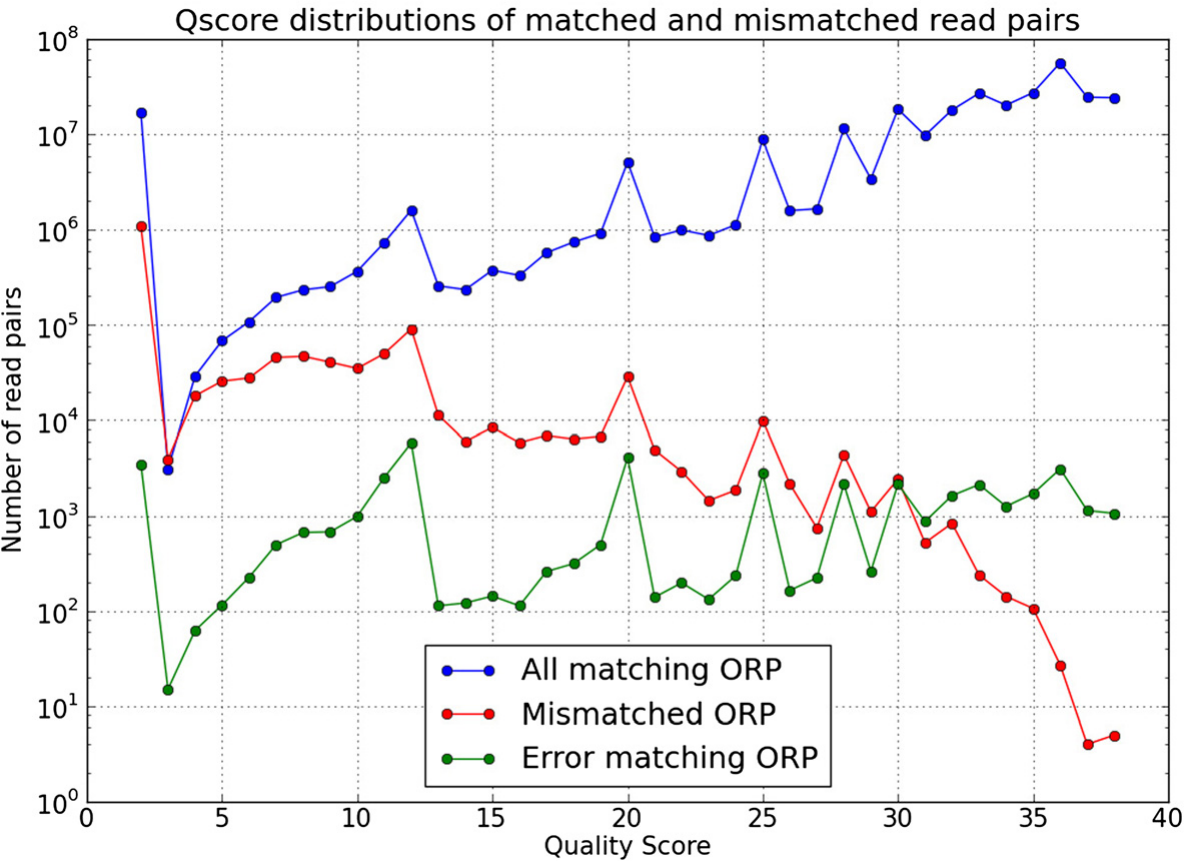
**Q-score distributions for all matching (blue), mismatched (red) and erroneous matching (green) overlapping read pairs in the BCV control sample.** Note, the Q-score of 2 is a ‘read segment control indictor’ in the FASTQ format that tags specific final portion of the read as unreliable and unfit for downstream analyses [41]. That Q=2 reads comprised a disproportionally large fraction of mismatched read pairs (red) is consistent with the fact that mismatched ORPs result from error during sequencing.

Figure 3 shows the Q-score distributions for all matching ORPs (blue), mismatched ORPs (red) and those matching ORPs that were erroneous (green) in the BCV control data (representative sample). Matching read pairs were labeled erroneous when they disagreed with the consensus nucleotides in the control plasmid. Consistent with Figure 2, the number of mismatched ORPs decreased with Q-scores while the number of matching ORPs increased with Q-scores. In contrast, the number of erroneous matching ORPs was relatively constant with respect to Q-scores, which suggests that they were dominated by PCR errors and not sequencing errors. Furthermore, Figure 3 shows that at Q ≥ 30, the number of mismatched ORPs (sequencing error) became dramatically lower than the number of erroneous matching ORP (PCR error). This provided an empirical guideline for the selection of Q-score threshold, above which PCR error rate clearly exceeds the sequencing error rate.

The two plasmid clones were sequenced at ultra-deep coverage to serve as controls in the study. At a given locus, any polymorphisms in the ORPs that deviated from the known consensus nucleotide are taken as errors introduced either through PCR amplification or sequencing. (Since only matching ORPs are considered here, these polymorphisms are erroneous matching read pairs.) The mean probability of such an error was estimated from the fraction of erroneous calls among all calls made across the control plasmid, which later served as the baseline error rate in our variant detection model. Another useful statistic is the maximum per base-call error rate – frequency of the erroneous base-call with the highest frequency in the control plasmid -- which provided an upper bound on the combined PCR and sequencing error that occurred at a single locus. Table 3 summarizes these error rates in the control plasmids at Q10 and Q30 for ORP and the two single reads that make up the read pairs. Notably, the maximum per base-call ORP error at Q10 and Q30 were comparable, which suggests this error was fairly stable and did not improve with Q-score. This result paralleled the finding that the frequency of erroneous matching ORP was relatively constant with Q-scores (Figure 3). Together, they suggest that the maximum per base-call errors observed in matching ORPs was likely produced by PCR.

**Table 3:** Summary of per base-call error rates in the two control sequences

**Read type**	**Control sample**	**Q10 mean error rate**	**Q10 max error rate**	**Q30 mean error rate**	**Q30 max error rate**
ORP	BCV	1.37×10^-4^	5.84×10^-4^	6.62×10^-5^	6.21×10^-4^
	Rabies	1.43×10^-4^	3.60×10^-4^	7.40×10^-5^	3.60×10^-4^
Forward reads	BCV	4.78×10^-4^	2.58×10^-2^	8.74×10^-5^	6.05×10^-4^
	Rabies	6.13×10^-4^	1.11×10^-2^	9.76×10^-5^	7.33×10^-4^
Reverse reads	BCV	4.95×10^-4^	9.32×10^-3^	9.44×10^-5^	5.88×10^-4^
	Rabies	6.70×10^-4^	5.00×10^-3^	1.06×10^-4^	1.48×10^-3^

Mean and maximum error rates at Q10 and Q30 are listed for matching ORP as well as the forward and reverse reads that make up the read pairs.

**Figure 4:**
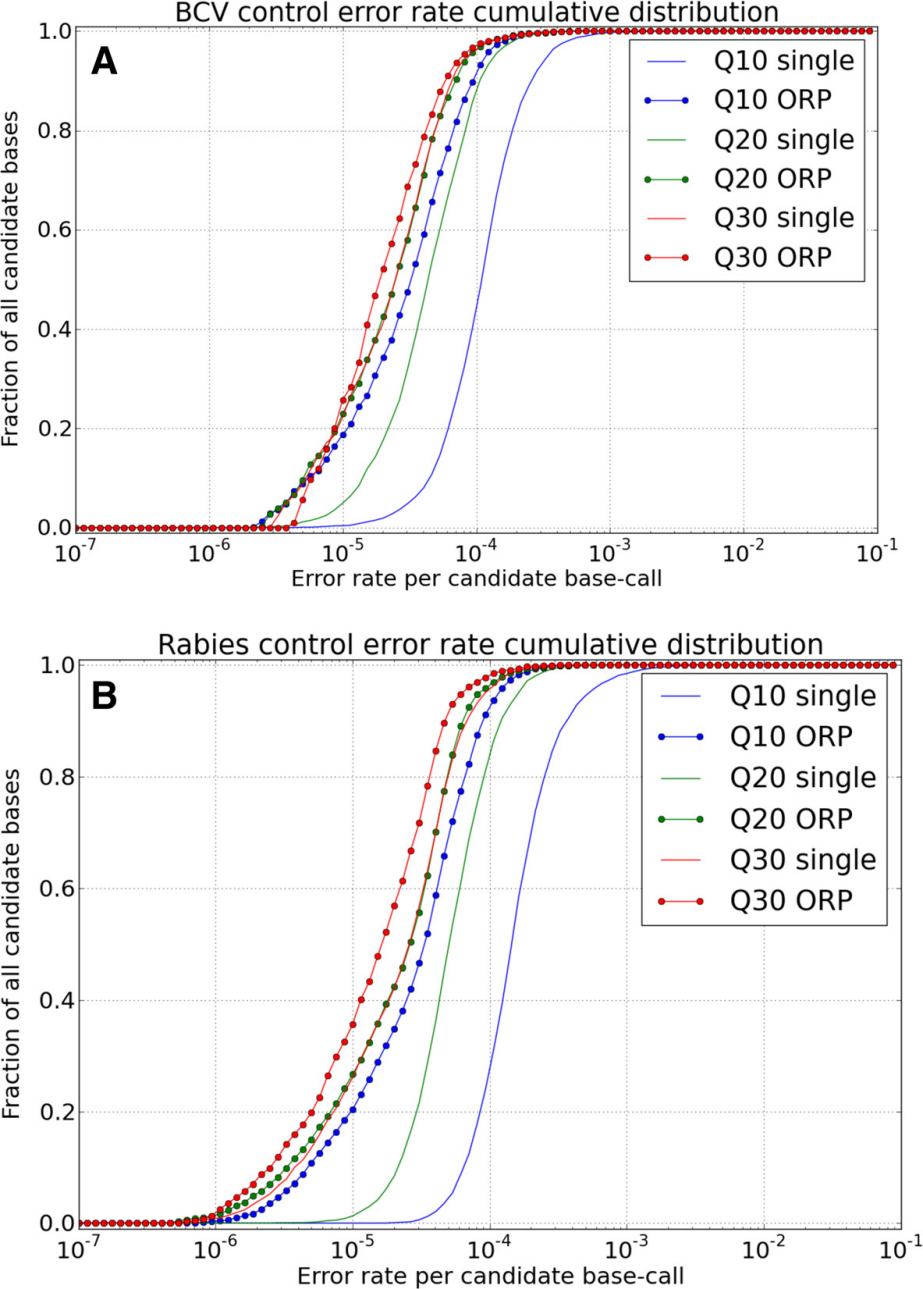
**Cumulative distributions of error rates in the two control data sets. A.** BCV control data. **B.** Rabies control data. Lines: single reads from reverse reads. Dotted lines: ORP reads.

As expected, matching ORPs have reduced error rates compared to the two single reads that make up each read pair. At Q10, this reduction is 3 fold in the mean error rate and one to two orders of magnitude in the maximum error rate (Table 3). Figure 4 shows the cumulative distribution of per base-call errors in the matching ORP and single reads thresholded at Q = 10, 20 and 30. The read accuracy improvement of using ORP over single reads, revealed as a gap between their respective error profiles, was most prominent at Q10 and diminished with higher Q-score filtering; the advantage at Q30 was small yet still highly significant (two-sample Kolmogorov-Smirnov test p = 5×10^-36^ and 2×10^-11^ for rabies and BCV control, respectively), as any reduction in error rate can be used to detect viral variants with greater sensitivity. In our data, error rates in Q30 single reads were equivalent with Q20 ORP reads. This equivalence, however, may not be generalizable to other data sets due to variations in Q-score metrics shown earlier (Table 2).

Our variant detection model uses a *position-dependent* error rate that takes the mismatch rate found at each base into consideration (Methods). Before applying this model on the natural samples, we applied it to the control data and determined that the false positive rates associated with 3 error rates, 5×10^-5^, 1×10^-4^ and 5×10^-4^ were 2.45%, 0.75% and 0%, respectively (Additional file 1: Table S2). This suggests that the mean Q30 ORP error rate of ~5×10^-5^ estimated from the control data (Table 3) was not conservative enough to eliminate all false positive variant calls, possibly due to PCR errors. Based on these observations, we made variant calls in the three natural viral samples using both 5×10^-5^ and a more conservative error rate of 5×10^-4^, where the former represented the mean overall error rate and the latter approximated the PCR error rate.

### Rare variants found at 10-13% of the genomes in the natural viral samples

Our variant detection model predicted 2133, 1354 and 1596 sub-consensus variants for the BCV, Fox1 and Fox2 natural viral samples, respectively (Table 4) using the error rate 5×10^-5^, and 152, 70 and 88 variants for BCV, Fox1 and Fox2 at the higher error rate of 5×10^-4^. The coverage-adjusted false positive rates for the error rates 5×10^-4^ and 5×10^-5^ are estimated to be 0% and < 2% (derived from running the model on the plasmid controls, Additional file 1: Table S2). Assuming a conservative false positive rate of 2%, then between 300 to 400 of the variants called at 5×10^-5^ are false positives, leaving a possibly true viral variant pool of 1724, 1063, 1187 variants for the BCV, Fox1 and Fox2 natural viral samples, respectively. These pools are over one order of magnitude larger than those predicted at the higher error rate of 5×10^-4^ and represent point mutations in 10-13% of the genomes.

**Table 4:** Summary of coverage, total number of candidate variants (all polymorphisms) and number variants called by the variant detection model in Q30 ORP for the 3 natural viral samples

**Natural sample**	**Mean ORP coverage**	**# candidate variant calls at Q**≥**30**	**# variants called at error rate 5**×**10**^**-5**^	**# variants called at error rate 5**×**10**^**-4**^	**FDR for error rate 5**×**10**^**-5**^	**FDR for error rate 5**×**10**^**-4**^
BCV	79,037x	20413	2133	152	19%	0%
Fox1	80,546x	14472	1354	70	21%	0%
Fox2	96,198x	20417	1596	88	26%	0%

The number of candidate variants served as the Bonferroni correction factors in the variant detection model. The number of false discoveries in each sample at error rate 5×10^-5^ is estimated by taking 2% (coverage adjusted false positive rate derived from control plasmids, Additional file 1: Table S2, ) of the number of candidate variants. The false discovery rate (FDR) is the proportion of false discoveries among variants discovered, e.g. for BCV, it’s the ratio between 2% of 20413 and 2133, i.e.19%. FDR at 5×10^-4^ is 0% for all samples since the estimated false positive rate at this error rate is 0% (Additional file 1: Table S2).

**Figure 5:**
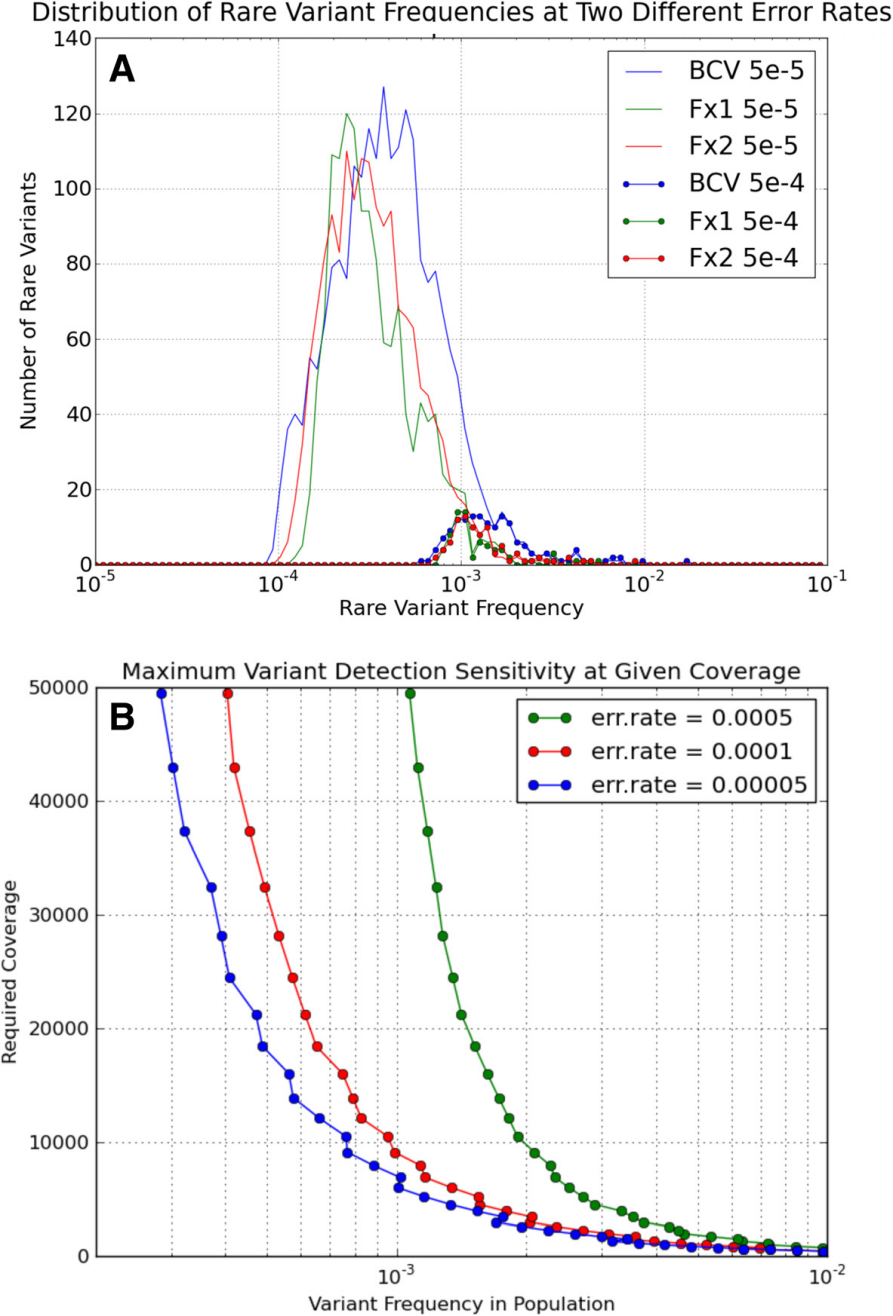
**Sensitivity of rare variant detection using ORP. A.** Distributions of rare variant frequencies detected in the three natural samples using two different error rates -- 5**×**10^-5^ (solid lines) and 5**×**10^-4^ (dotted lines) in the binomial variant detection model. **B.** Theoretical maximum variant detection sensitivity at given coverage level for three different error rates. Coverage level is for usable reads that exhibit the error rate under consideration.

The distributions of the rare variant frequencies provide additional insight to the dramatic difference in the number of variants called at these two error rates. Figure 5A shows that most of the variants detected at 5×10^-5^ were far more rare than the least frequent variants detected at 5×10^-4^. This means, the higher error rate of 5×10^-4^ was not sensitive enough to detect the majority of the mutations detected at the lower error rate of 5×10^-5^, which existed at frequencies below 0.05%.

Recovering the bulk of these ‘ultra-rare’ viral mutations, however, requires considerable extra effort – either through reduced error rate or increased coverage. Since for most viral evolution study designs it may not be practical or necessary to sequence viral samples at the coverage level used in this paper, we estimated the theoretical coverage required to achieve specific variant detection sensitivity under three error rates: 5×10^-5^, 1×10^-4^, and 5×10^-4^. Predictions are based on a binomial error model with a fixed Bonferroni correction factor (assuming 11,000 candidate variants detected in every case) and shown in Figure 5B. For instance, at 20,000x coverage (matching ORP), the maximum sensitivity, or the rarest variants that can be detected at these three error rates have population frequencies of 0.05%, 0.065% and 0.145%, respectively, with the lowest error rate (5×10^-5^) being the most sensitive and the highest error rate (5×10^-4^) being the least sensitive.

As shown in Figure 4, accuracy of Q30 single reads is comparable to that of Q20 ORPs. In the scenario of 20,000x ORP coverage, if non-overlapping read pairs had been generated during sequencing, in theory, the number of Q30 single reads could potentially double the coverage to 40,000x. At the error rate of 5×10^-5^, the maximum sensitivity for variant detection in these Q30 single reads would be 0.03% instead of 0.05%. The cost of this increased coverage by generating non-overlapping read pairs is losing the benefit of context-specific error estimation and correction afforded by the overlapping read pairs. The accuracy comparison between Q20 ORPs and Q30 single reads observed in our data set was only established because ORPs were used. Any accuracy equivalence between single reads and ORP has to be carefully re-established for the specific sequencer being used.

## Conclusions

In order to effectively use ultra-deep sequencing to study rare members of a viral population, it is critical to accurately model errors in the sequencing data. We have developed a protocol to evaluate and control sequencing error in multiple ways. First, plasmid clones were sequenced along with viral samples of interest so that error rates in the PCR and sequencing process could be empirically derived. Second, taking advantage of the Illumina paired-end technology, overlapping paired-end reads were generated to improve read accuracy. Third, mismatch rates in the ORP and error rates in the control plasmids were examined in association with quality scores from the sequencers so an optimal Q-score threshold could be selected for read filtering. Mismatch rates were also incorporated in the variant detection model to dynamically adjust error rates based on local sequencing errors. Applying this model on the plasmid control data before the natural samples further gave empirical assessment of false positive rates in the data for a given error rate.

Sequencing errors were directly estimated from mismatch rates in the ORP. We found that these Q-score filtered ORPs had sequencing errors far below what their Q-scores suggested. Using mismatch rates in the ORP, we demonstrated considerable variability of quality metrics on two Illumina GA IIx sequencers (Table 2). This variability may stem from differences in the Q-score calibration process on the sequencers. Together, these findings suggest that Q-scores by themselves are not reliable measures of sequencing accuracy. Mismatch rates in ORPs, however, provide unbiased estimates on sequencing error and can be used to select Q-scores for read filtering.

Among the challenges of correctly separating the true mutations from sequencing related errors is the presence of the non-uniform error rates in the sequencing data [15,20-22]. We have shown that even at Q30, ORP mismatch rates have a small but significant distribution (Figure 2). Mismatch rates in the ORPs offer locus-specific information on error rates and thus can improve variant call accuracy. Besides the Illumina paired-end technology, repeated measurements on the same read fragments can also be generated using other sequencing platforms such as the PacBio RS from PacificBiosciences [30]. As with techniques developed in this work, future new data generation methods that support high-throughput repeated interrogation of the same insert fragment and informatics techniques that exploit this feature can also be used in combination to substantially reduce the possibility of error.

ORP has potentially greater benefits in the application of direct sequencing without PCR. Our results show that with careful quality control, the accuracy of PCR-amplicon sequencing will be limited by PCR rather than sequencing errors. While it is often necessary to use RT-PCR to amplify viral RNA from host material, the exponential nature of the PCR reaction combined with issues such as primer bias can skew the variant frequencies (e.g. toward laboratory-derived reference strains) and even generate incorrect consensus sequences in some regions of the genome. This highlights the importance of pursuing alternative, direct sequencing technologies. Until recently, it has not been practical to sequence viral samples such as ours without PCR amplification. As the technology of direct sequencing improves and becomes available, methods that can reduce sequencing error, such as the use of ORP, will play a greater role in quality control.

The tradeoff for enhanced accuracy by using ORP and Q-score filtering is the reduction in coverage. Choosing to generate overlapping read pairs instead of non-overlapping read pairs can reduce the effective coverage by half. Another limitation of ORPs is the effective shortening of read length, which limits downstream analysis such as linking multiple sub-consensus mutations to a single haplotype. Nevertheless, currently the longest length of non-overlapping read pairs is still too short to span an entire gene or genome for the purpose of reconstructing subconsensus haplotypes.

Several alternative ultra-sensitive mutation detection approaches have been proposed recently for next generation sequencing [15,31,32], but they require significant sample preparation and may not be practical beyond targeting limited regions of a genome. Both Duplex Sequencing [32] and Safe-SeqS [31] require labeling the DNA fragment libraries with unique sequence tags (UID) prior to PCR amplification. Post-sequencing, mutations that occur in the majority of their uniquely tagged read families are identified as true variants. These methods successfully address the errors introduced during PCR and sequencing but are subject to the efficiency of the UID assignment. A significant fraction of the starting material is generally lost in the library prep procedures for Illumina sequencing due to poor adapter ligation efficiency and the requirement of multiple clean-up cycles. If a sample contains limited starting material for sequencing, as is often the case in viral or clinical samples, performing adapter ligation prior to PCR amplification will likely lead to poor representation of the sample. Furthermore, the additional UID assignment process adds to the complexity and cost of sample preparation. Thus while these methods present a possible approach for the future, their scalability to whole genome sequencing has not yet been demonstrated. Flaherty et al. [15] proposed an ultrasensitive mutation detection method for targeted resequencing using a position-specific error profile. They derive the position-specific error profile of a 700 bp region of the NA gene in H1N1 using a clone of the sample of interest. This approach while highly specific, also does not scale to whole genome analysis. In contrast to these methods, we suggest that the use of ORPs to derive error profiles is easily extendible to a genome of any size without the use of a cloned sample. Comparable fragment length and read length is the only requirement to generate ORPs.

This study for the first time provides a detailed analysis of the current standard single read approach versus overlapping read pairs in the application of mutant spectra profiling of viral samples. The results show there are compelling benefits to using overlapping read pairs, which lead us to favor their use whenever feasible. Yet the results also show that with careful filtering, single reads provide a viable option when PCR amplification is used in the sample preparation stage, *and* enough experience with the specific sequencer is available to eliminate the impact of unexpected changes in calibrated quality scores or other technical configuration changes. The advantage of ORPs is the dynamic context specific error estimation and error correction, which significantly reduces sequencing error and is more robust in the face of configuration changes across sequencing machines.

Sensitivity of viral variant detection is a function of error rate and coverage. We provided theoretical maximum sensitivity for a given coverage at three different error rates (Figure 5B). Consistent with these predictions, the majority of the variants detected in our data occurred at the frequencies of 0.01% to 0.1% for the error rate of 5×10^-5^. For our samples, the ‘ultra-rare’ mutants make up a majority of the sub-consensus population (Figure 5A). These ultra-rare mutants greatly increase the genetic diversity in the quasispecies and may play important roles in acute viral infectious diseases. While it remains an open question as to their ultimate importance for downstream applications, our results illustrate the significance of reduced error and increased coverage in recovering these rare mutations. Reliable rare variant detection and sequence error reduction is important for many research areas beyond virology. Our methods have applications in clinical diagnostics [15,31], forensics [33] and DNA-based information storage system [34]. Although high-throughput sequencing technologies have been limited in some applications due to their high error rates, repeated template sequencing presents a powerful approach for increasing the sequencing fidelity to a level that can generate highly sensitive detection assays.

## Methods

This study was approved by the Institutional Animal Care and Use Committee at Lawrence Livermore National Laboratory (Protocol Number 2009–207). The rabies positive brain samples included in this study were not covered by IACUC 2009–207 as they were taken from residual archived diagnostic samples offered by the California Department of Public Health Laboratory, Richmond, CA and were originally collected and tested to inform public health decisions on administering anti-rabies vaccination.

The data described in this paper is available via anonymous ftp as reads, BAM format alignments, consensus sequence, nucleotide frequency profiles at: ftp://gdo144.ucllnl.org/pub/orpdat/.

### Virus samples

Rabies: Two brain tissue samples obtained from grey foxes (*Urocyon cinereoargenteus*) displaying symptoms of rabies were collected in Humboldt County, CA in March 2009 and December 2009 and tested for rabies virus via RT-PCR [35] using a modified protocol that amplifies a portion of the N gene. Approximately 1 gram of tissue from each brain was sent to LLNL for analysis. RNA was extracted from the tissue sample using TRIzol® LS Reagent (Invitrogen, Carlsbad, CA) following the manufacturer’s protocol.

Bovine coronavirus (BCV): Nasal samples were collected from approximately 100 asymptomatic calves. Samples were collected using sterile polyester swabs, placed in 2-3 mL of Eagle’s Minimum Essential Medium (Gibco) and transported on ice back to the laboratory. Collected nasal swabs were vortexed in Eagle’s Minimum Essential Medium supplemented with 1% antibiotic-antimycotic solution (Gibco). The sample suspensions were clarified by centrifugation at 2000 x g for 30 minutes, filtered through a 0.22 μm filter and aliquots of about 500–1000 μl were stored at −80°C. RNA was extracted using TRIzol® LS Reagent following the manufacturer’s protocol. Samples containing BCV RNA were identified as described in Cho et al. [36].

### Genome amplification

Approximately 11 kb of the rabies virus genome and 12 kb of the BCV genome were amplified using reverse transcriptase (RT) PCR. PCR primer candidates were selected based on the combined results of the multiple sequence alignment and sequence searches. This technique is a modified version of the approach outlined in Slezak et al. [37]. Three sets of degenerate PCR primers were tested for the amplification of each overlapping region of the rabies virus and BCV genomes using increments of 1.5-2.5 kb. For each region, the two primer sets that performed best were used to amplify cDNA obtained from the two fox rabies samples and one BCV sample. All primers used for amplification are given in Additional file 1.

### RT-PCR and cloning

The rabies and BCV genomes were amplified using two-step RT-PCR using Superscript III RT reverse transcriptase kit and Platinum Pfx polymerase (Invitrogen), following manufacturer's instructions. Reverse transcription was performed using random hexamers the PCR conditions consisted of 94°C for 5 min, followed by 35 cycles of 94°C for 15 s, 54-60°C for 30 s, and 68°C for 2.5 min.

A 1 kb region of the rabies virus and BCV genome were amplified and each cloned into a plasmid vector. The inserts were generated by RT-PCR as described above using rabies and BCV polymerase primers: RVpolyF1 5’ CCCCTGACTCCTTATATCAAAACC, RVpolyR1 5’ GCGAGGTTGACTATTTGGTC, BCVpolyF2 5’ TTTGCAGACAAATTGGTGGA, and BCVpolyR2 5’GGCGTAAATTTCATCCTGCT. Poly 3’ A overhangs were added to the PCR products by incubating the products with Taq polymerase at 72°C for 10 min. TOPO TA Cloning Kit for Sequencing (Illumina) was used to clone the PCR products into One Shot® TOP10 cells (Invitrogen) as per manufacturer’s instructions. Sanger sequencing of the cloned controls was carried out by ELIM Biopharmaceuticals, Inc., Hayward CA. PCR products were prepared for Illumina sequencing using the QIAquick PCR Purification kit (Qiagen).

### Illumina sequencing

Sequencing of the three natural samples and the two control plasmids was carried out by Eureka Genomics (Hercules, CA) using an Illumina Genome Analyzer IIx. Each natural viral sample was sequenced in a separate lane of a single flow cell using paired-end reads on short genomic fragment inserts using read lengths of 112 bases. The clonal controls were mixed in a single sample with an approximate concentration ratio of 10:1 (rabies:BCV) and sequenced on a separate lane. Since the PCR primers could potentially introduce false mutations into the amplicon pool due to non-specific binding, primer regions were masked out for the downstream analysis. Table 1 summarizes the output generated in the sequencing runs.

To compare error rates between different sequencers, rabies control plasmid was sequenced a second time at Elim Biopharm (Hayward, CA) using Illumina Genome Analyzer IIx. A shorter fragment length was chosen with more complete overlap of the read pairs. The same amplicon pool was used for both sequencing runs.

### Read mapping to reference

The open source software SHRiMP2 was used for read mapping. The tool was chosen for its high read mapping sensitivity [38] and its ability to map as many reads as possible in the face of individual errors within each read [39].

A consensus sequence was generated for each sample following an iterative comparative assembly procedure suggested by Willerth et al. [16]. In this approach, an initial reference sequence was chosen, reads were mapped to the reference, then a new consensus sequence was generated and the reads were mapped to the new consensus again. The procedure continued until the consensus converged on a single sequence.

All rabies reads were initially mapped to GenBank rabies reference sequence GI:260063801. This reference sequence was used as the common coordinate system for comparing samples and identifying coding frames. Similarly, GenBank bovine coronavirus GI:15081544 was used as the reference sequence for the BCV samples.

Based on a later observation that our sequenced rabies virus genome differed by approximately 9% relative to the pre-selected reference fox rabies genome, we checked to see if observed error rate (defined below) would increase by introducing random mutations at 9% of the plasmid control reference sequences generated from Sanger sequencing. Increased divergence between the sample and the randomly mutated reference sequence could confound the read mapping program and introduce additional alignment errors, however, no noticeable increase in error rates were observed, suggesting that the read mapping parameters were able to tolerate this rate of divergence.

### Estimation of error rate from control plasmids

The two plasmid controls were used to empirically model combined PCR and sequencing errors as well as to evaluate our algorithm for making genetic variant calls. The clone control samples were amplified using the same PCR amplification protocol as was used for sequencing natural samples. A control reference sequence was generated from a separate Sanger sequencing run. Any polymorphisms that deviated from the consensus sequence were taken to be examples of error introduced either through PCR amplification or sequencing.

At every base, any nucleotide called by a read is referred to as a “candidate base call”. Error rates were calculated as a ratio between the total number of candidate base calls differing from the consensus nucleotides summed across the genome and the total number of base calls made across the genome.

### Quality control of the sequencing data

The following rules were implemented to maximize the quality of the reads. Quality scores (Q-score) from the sequencer were used to compile nucleotide frequency distribution for every base sequenced. These nucleotide frequency distributions were generated at four quality thresholds for comparative analyses: raw (all reads), Q≥10, Q≥20 and Q≥30. At a given base, a read covering the base contributes to a candidate base call only when the minimum Q-score over an 11-nucleotide window (±5bp) centered on the query base surpasses the quality-score threshold being considered (e.g. the Q30 nucleotide frequency profile). In addition to Q-score filtering, a misalignment filter required that the 11-nt window contained no indels and that the query position must be at least five bases away from the end of the read to avoid misalignment for single reads. To avoid the potential of higher error rates at the 3-prime ends of single reads, only the first 80 bases were used in single read analyses. Furthermore, when calculating error rates and making variant calls, only those bases where greater than 10% of the reads that survive the quality filters are considered for analysis at that quality score. This is to avoid inclusion of non-representative features for select bases in the genome.

### Sequencing error analysis

At every base, all overlapping read pairs were separated into two categories: matching and non-matching base pairs. Matching base pairs have two complementary nucleotides and non-matching base pairs have two incongruent nucleotides. Mismatched read pairs were only used to calculate mismatch rates and examine the relationship between mismatch rates and quality scores. Other than that, mismatched read pairs were excluded from all error and variant analyses.

Mismatch rates were calculated two ways, ‘per position’ and ‘per base pair’. Per-position mismatch rate is the fraction of overlapping read pairs that are mismatched at any given location in the genome. Per base pair mismatch rate is the total number of mismatched read pairs summed across all bases in the genome divided by the total number of read pairs.

To generate quality score distributions for the matching and mismatched read pairs, Q-scores for every base pair were compiled as follows. For a matching base pair, the average quality score was used. For a non-matching base pair, the minimum quality score was used. The resulting Q-score distributions were compared and used to generate Q-score receiver-operator characteristic (ROC) and ‘false discovery rate’ curves.

Sequencing errors can occur in two forms in overlapping read pairs. Non-complementarity between the forward and reverse strands at a given base indicates that at least one of the two nucleotides is erroneously incorporated. This type of error is straightforward to exclude -- all non-matching read pairs are excluded from analysis except in the analyses for quality control. A second, more rare but ‘hidden’ form of error is where two complementary errors occur on both the forward and the reverse strands such that the resulting read pair remains complementary.

### Making variant calls

Variant calls were made based on the binomial error model used by Eriksson et al. [40], with modifications to address the non-uniformity in sequencing error rate measured with ORP mismatch rates. Specifically, the probability of observing x or more mutations in N matching read pairs covering the base is given by the survival function of the binomial distribution *B (N, p*)

$$\mathrm{PP}\left(X\;\geq \;x\right)=\sum_{k=x}^{N}\left(\begin{array}{c} N\\ k \end{array}\right)p^{k}{\left(1-p\right)}^{N-k}$$

The error rate, *p*, is the combined PCR and sequencing error, *ε*, adjusted by a function of the ORP mismatch rate, *δ*, at the base in question.

The read-pair mismatch rate, *δ*, is the *position-dependent* rate at which a nucleotide is mis-incorporated into a single strand. For simplicity, we modeled the probability where two complementary nucleotides are mis-incorporated simultaneously on forward and reverse strands at the same base as *δ*^2^. We used the maximum of the clonal control derived error and the square of the ORP mismatch rate at a particular base as the adjusted *position-dependent error rate* for binomial error model

$$p=\max\left(\varepsilon ,\delta^{2}\right)$$

P-value = 0.01 with Bonferroni correction was used as the significance threshold for each hypothesis test.

Scripts for the variant detection model were written in Python and R and are available upon request.

## Competing interests

Authors declare that they have no competing interests.

## Authors’ contributions

HC and JEA carried out the sequencing analysis and drafted the manuscript. JEA and MB conceived of the study, developed the sequencing protocol, designed and coordinated the experiments. CT and TRS carried out the primer design. All authors read and approved the final manuscript.

## Supplementary Material

Additional file 1:Figure S1, Table S1, Table S2, Table S3, Table S4 Figure S1 is on the effect of quality filtering on coverage. Table S1 summarizes the sequencing output. Table S2 lists the false positive rates among ORP reads after applying the variant detection model to the control sequences at their coverage level and two other hypothetical coverage levels. Table S3 and table S4 list the primers used to amplify regions of the BCV and rabies genomes, respectively. Additional discussion on the relative contribution of sequencing and PCR error rates is also included. Click here for file
