# Supplementary material for: Ultra-deep mutant spectrum profiling: improving sequencing accuracy using overlapping read pairs
Source: BMC Genomics. 2013 Feb 12;14:96. doi: 10.1186/1471-2164-14-96 (PMC3599684; doi:10.1186/1471-2164-14-96)
Supplement: Additional file 1:Figure S1, Table S1, Table S2, Table S3, Table S4 — Figure S1 is on the effect of quality filtering on coverage. Table S1 summarizes the sequencing output. Table S2 lists the false positive rates among ORP reads after applying the variant detection model to the control sequences at their coverage level and two other hypothetical coverage levels. Table S3 and table S4 list the primers used to amplify regions of the BCV and rabies genomes, respectively. Additional discussion on the relative contribution of sequencing and PCR error rates is also included. [file 1471-2164-14-96-S1.pdf]

## Additional information

### Effect of quality filtering on coverage

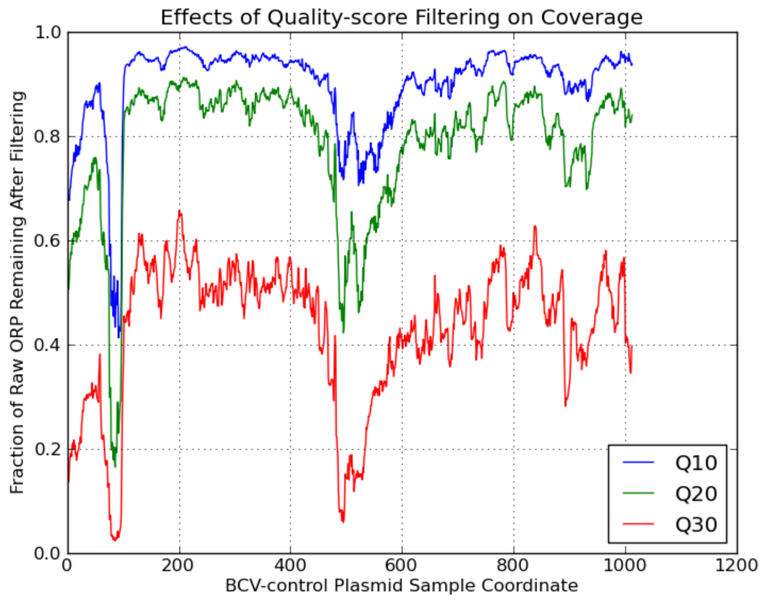


**Figure S1. Coverage of the BCV control plasmid by matching ORP reads thresholded at three quality scores, Q10 (blue), Q20 (green) and Q30 (red). Coverage at any base in the plasmid is shown as fraction of the raw matching ORP reads that remains after Q-score filtering.**

The trade-off for enhanced accuracy is the reduction in coverage. Figure S1 shows the coverage of the BCV control plasmid by matching ORP as fractions of all matching ORP coverage produced by the sequencer. Compared to the raw matching ORP coverage of 284,803x per base, the average coverage at Q10, Q20 and Q30 are 252,419x (88.6% of raw), 221,070x (77.6%) and 128,942x (45.3%) per base, respectively.

### Relative contribution of sequencing and PCR error rates at Q10 vs. Q30

A direct assessment of the error contribution can be made by comparing Table 3 with ORP mismatch rates given in Table 2. At Q10, the mean ORP mismatch rates in the two control plasmids are over $7\times10^{-4}$, greater than either the mean or the maximum error rates in matching ORP (proxy for upper bound on PCR error), suggesting sequencing error in Q10 single reads is likely a greater source of error than PCR. At Q30, however, the mean ORP mismatch rates drop to near $2\times10^{-6}$, significantly lowering the contribution of sequencing error to the overall error rate.

We initially hypothesized that the use of high fidelity polymerase would minimize the impact of PCR errors compared to sequencing errors. But our results show that PCR is a major source of error. The relative contributions of these two sources to the overall error rate change with respect to Q-scores and can be clearly demonstrated in the control plasmid sequences. This highlights another benefit of using ORP in combination with a

sequencing control plasmid -- to provide empirical measurement on PCR error rate and optimal Q-score selection that guarantees a lower average sequencing error rate than the PCR error rate. In the absence of a sequencing control plasmid, the ORP mismatch rate can be used to identify a Q-score threshold that corresponds to some predetermined, acceptable sequencing error rate that takes into account the amount of available coverage and the target detection sensitivity. Thus, the ORP mismatch rate can take the place of a control.

**Table S1. Summary of sequencing output.**

| Sample | Sequence output (gigabases) | Number of Reads |
|---|---|---|
| Plasmid controls | 6.3 | 2 x 28,325,049 |
| Rabies 1 | 6.06 | 2 x 27,063,566 |
| Rabies 2 | 6.06 | 2 x 27,051,934 |
| Bovine coronavirus | 6.1 | 2 x 27,231,827 |

**Table S2. False positive rates among ORP reads after applying the variant detection model to the two control sequences at three different error rates. Shaded rows show for the coverage the plasmids are sequenced, the number of type I errors that survived the Bonferroni correction and, in parenthesis, the corresponding false positive rate, i.e. their percentage among all candidate variant calls in the sequencing results. The number of type I errors that would have been found to be significant is also estimated at two other coverage levels: 50,000x and 5000x.**

| Sample | ORP coverage level | # type I errors (FP rate) using error rate $5\times10^{-5}$ | # type I errors (FP rate) using error rate $1\times10^{-4}$ | # type I errors (FP rate) using error rate $5\times10^{-4}$ |
|---|---|---|---|---|
| BCV-control | 128,942x | 30 (1.7%) | 12 (0.7%) | 0 (0%) |
| | 50,000x | 15 (0.9%) | 6(0.4%) | 0 (0%) |
| | 5000x | 0 (0%) | 0 (0%) | 0 (0%) |
| Rabies-control | 773,021x | 66 (3.2%) | 17(0.8%) | 0 (0%) |
| | 50,000x | 6 (0.3%) | 1 (0.05%) | 0 (0%) |
| | 5000x | 0 (0%) | 0 (0%) | 0 (0%) |

**Primers used to amplify BCV and rabies genome:**

| Identifier | Sequence (5' to 3') |
|---|---|
| A_FOR_2_2616_BCV | TGTGGTTATTCTGAACCACCTAAAGTTGCA |
| A_FOR_3_2652_BCV | ATTTGCATTGTGGATAATGTTTATATGGCC |
| A_REV_2_4797_BCV | CGGGACACTCAAAATACTTAACGG |
| A_REV_3_4854_BCV | AACTACCCTGCTGTACATAACCAAA |
| B_FOR_1_4600_BCV* | TAAGCAATGACGTTGCATTTGTTTCG |
| B_FOR_2_4643_BCV | GGATGTTTTATCCTTAAGACATGATATAGC |
| B_FOR_3_4674_BCV | CTTGATGATGATGCACGAACCTTT |
| B_REV_1_6519_BCV* | ACTTACGTATTGTAGGTACGTTAACTGC |
| B_REV_2_6542_BCV | AAGAGTCATACCAAATTTTATAAACTTACG |
| B_REV_3_6580_BCV | ATCTCTCTTAAATTTAACAAATCAATTGGT |
| C_FOR_2_6344_BCV | AAAACCATTTAAGGTTGAAGATAGTGTCAT |
| C_FOR_3_6390_BCV | AGTGAAATCAAATATGTTAAGAGTTTGTCT |
| C_REV_2_8570_BCV | AAGCTGAAAATCTGATTTGTGTACTGTGTA |
| C_REV_3_8625_BCV | CGCTAACATCTCTAATAACACCATTATCTA |
| D_FOR_1_10700_BCV | AGCTTCTATGACTGGTGTGTCTTTGGA |
| D_FOR_2_10755_BCV | AAGAATGGTTTTCAAGGACGTCAGAT |
| D_FOR_3_10765_BCV** | TTCAAGGACGTCAGATTATGGGTAGTT |
| D_REV_1_11783_BCV | AACATGCAAATGTTGCAAGCAATTAAGCAA |
| D_REV_2_11836_BCV | AGTATTTCATTGTGCAAAGTGCTACAATA |
| D_REV_3_11743_BCV** | GCACATTTAACATCAGTCAATTTTGATTGA |
| E_FOR_2_17743_BCV | TGCAGAAACAGCGCATTCTGTAAATGTTAA |
| E_FOR_3_17764_BCV | AAATGTTAATCGCTTCAATGTTGCTATTAC |
| E_REV_2_19532_BCV | CACCAGCCTGTCCTGTATAATGACCAGT |
| E_REV_3_19545_BCV | GCACAAGGCATTTCACCAGCCTGTC |
| F_FOR_2_23542_BCV | CTTTTGGGTGTTGCGGTCATAATTATTGTA |
| F_FOR_3_23588_BCV | TTATGGTGGATAATGGTACTAGGCTGCAT |
| F_REV_2_25077_BCV | AACAATGTTGTGCATAAACAACATCATGAT |
| F_REV_3_25124_BCV | CAAAGACCCATCCAATTTACACGGACAGA |
| G_FOR_2_24931_BCV | ATAATTTACCTGCTGCTAATGTTTCTGTTA |
| G_FOR_3_24969_BCV | AATCCTTCTACTTGGAATAGGAGATTTGG |
| G_REV_2_26452_BCV | CAATTATTATAAGCCTCAACGAAACCGACA |
| G_REV_3_26437_BCV | TCAACGAAACCGACATCAGATAACTTT |
| H_FOR_1_26204_BCV | CACAGAAGTAAATGAACTACTTGACACTAC |
| H_FOR_2_26222_BCV | ACTTGACACTACACAGTTGCAAGTAGCTAA |
| H_REV_1_27837_BCV | TGTGGTAGCTATTATAATATGCTCGACCT |
| H_REV_2_27852_BCV | CTAAACAGCAGGCATTGTGGTAGCTATTAT |

Table S3. Primers used to amplify regions of the rabies genome.

| Identifier | Sequence |
|---|---|
| A_FOR_2_130_RAB | AGACTGGACCAGCTATGGAATCYTG |
| A_FOR_3_74_RAB | GATGTTGYTCYTACTTGGCAGC |
| A_REV_2_2209_RAB | TCATCTTCARCTGCTCAAAATTATACAAGA |
| A_REV_3_2159_RAB | GGAAAYTTGTACTTCTTGGARAAGCT |
| B_FOR_1_2056_RAB | CCAGCCCTWGARTGGTCTG |

| | |
|---|---|
| B_FOR_2_2099_RAB | CTGTAGAGGCWGAGATMGCTC |
| B_REV_1_3622_RAB | CTTTTGAAYGTGGTKGTGACA |
| B_REV_2_3687_RAB | GGTCGCCWGCCATYTTCCA |
| C_FOR_2_3594_RAB | GTTGGTTATGTCACMACCAC |
| C_FOR_3_3516_RAB | GGATACATCTCWKCCATAAAAGTGAACGG |
| C_REV_2_5641_RAB | TTTCAGAACTTTGTAAGACCKRGA |
| C_REV_3_5590_RAB | AGGTCTATTTCCTGTTYTCAVCC |
| D_FOR_1_5420_RAB | CCAGGAGAAGTYTATGATGAYCC |
| D_FOR_2_5568_RAB | GGTTGARAACAGGRAATAGACCT |
| D_REV_1_6742_RAB | CCATGTCTGGGTTTTGATATAAGG |
| D_REV_2_6757_RAB | TATATGTTTKGGWGGCCATGTCTGGGTTTT |
| E_FOR_1_6506_RAB | GGTTGTTACAGGCATTGGGG |
| E_FOR_2_6606_RAB | ACCAGGAGTGTTTAGCRAGYGA |
| E_REV_1_8291_RAB | GAAGGCGWGTGAAGCTYTC |
| E_REV_2_8281_RAB | GAAGCTTTCVAGTGTTCTCTCTCC |
| F_FOR_1_8108_RAB | GGKGTGTCTGGAATGTCTCT |
| F_FOR_2_8036_RAB | ATTYTGAGTGCTGAWGGGGA |
| F_REV_1_9741_RAB | TTGATGTTGATRTTTGTCATTCTTGTYA |
| F_REV_2_9660_RAB | TCTCTMGGGGAGACYTTGC |
| G_FOR_1_9641_RAB | AGCAARGTCTCCCCKAGA |
| G_FOR_2_9503_RAB | CCAGGTGATTTTGARTCTCTAARTG |
| G_REV_2_11142_RAB | CGRGCYCTCTGCATCTCACT |

Table S4. Primers used to amplify regions of the rabies genome.
